# Strange Attractors Generated by Multiple-Valued Static Memory Cell with Polynomial Approximation of Resonant Tunneling Diodes

**DOI:** 10.3390/e20090697

**Published:** 2018-09-12

**Authors:** Jiri Petrzela

**Affiliations:** Department of Radio Electronics, FEEC, Brno University of Technology, 601 90 Brno, Czech Republic; petrzelj@feec.vutbr.cz

**Keywords:** chaos, Lyapunov exponents, multiple-valued, static memory, strange attractors

## Abstract

This paper brings analysis of the multiple-valued memory system (MVMS) composed by a pair of the resonant tunneling diodes (RTD). Ampere-voltage characteristic (AVC) of both diodes is approximated in operational voltage range as common in practice: by polynomial scalar function. Mathematical model of MVMS represents autonomous deterministic dynamical system with three degrees of freedom and smooth vector field. Based on the very recent results achieved for piecewise-linear MVMS numerical values of the parameters are calculated such that funnel and double spiral chaotic attractor is observed. Existence of such types of strange attractors is proved both numerically by using concept of the largest Lyapunov exponents (LLE) and experimentally by computer-aided simulation of designed lumped circuit using only commercially available active elements.

## 1. Introduction

A general property of chaos is long-time unpredictability; i.e., random-like evolution of dynamical system even if the describing mathematical model does not contain stochastic functions or parameters. Because of its nature, chaotic behavior was often misinterpreted as noise. The first mention of this kind of the complex solution was in [1] where Lorenz noticed the extreme sensitivity of autonomous deterministic dynamics to tiny changes of the initial conditions. After this very milestone, chaos started to be reported in many distinct scientific fields as well as daily life situations. Chaotic motion has been observed in chemical reactions [2], classical mechanics [3], hydrodynamics [4], brain activity [5], models of biological populations [6], economy [7] and, of course, in many lumped circuits.

Two basic vector field mechanisms are required for evolution of chaos: stretching and folding. The first mechanism is responsible for exponential divergence of two neighboring state trajectories and second one bounds strange attractor within a finite state space volume. Pioneering work showing the presence of robust chaotic oscillation within dynamics of simple electronic circuit is [8]. So far, the so-called Chua´s oscillator was subject of laboratory demonstrations, deep numerical investigations and many research studies [9,10,11]. Several interesting strange attractors associated with different vector field local geometries have been localized within the dynamics of three-segment piecewise-linear Chua systems [12,13,14]. However, the inventors of Chua´s oscillator built it intentionally to construct a vector field capable of generating chaotic waveforms. Progress in computational power together with development of the parallel processing allows chaos localization in standard functional blocks of radiofrequency subsystems such as in harmonic oscillators [15,16], frequency filters [17,18], phase-locked loops [19], power [20] and dc-dc [21] converters, etc. From a practical point of view, chaos represents an unwanted operational regime that needs to be avoided. It can be recognized among regular behavior because of the specific features in the frequency domain: continuous and broadband frequency spectrum. However, an approach that is more sophisticated is to derive a set of describing differential equations and utilize the concept of LLE to find regions of chaotic solutions [22].

Searching for chaos in a mathematical model that describes simplified real electronic memory block is also topic of this paper. Three programs were utilized for the numerical analysis of MVMS: Matlab 2015 for the search-through-optimization algorithm including CUDA-based parallelization, Mathcad 15 for graphical visualization of results and Orcad Pspice 16 for circuit verification. The content of this paper is divided into four sections with the logical sequence: model description, numerical analysis, circuit realization and verification; both through simulation and measurement.

## 2. Dynamical Model of Fundamental MVMS

**Figure 1:**
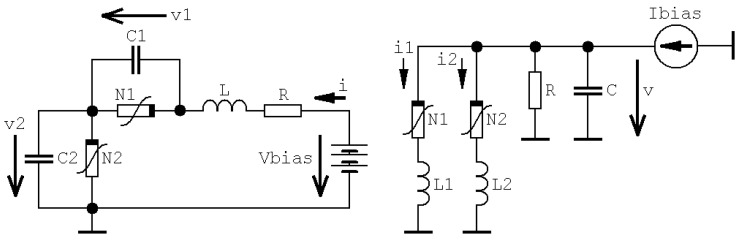
Basic network configurations of MVMS: original (**left** schematic), dual (**right** schematic).

Basic mathematical model of MVMS [23,24] is given in Figure 1 and can be described by three first-order ordinary differential equations in the following form:

(1) $$C_{1}\frac{dv_{1}}{dt}=i-f_{1}(v_{1})\;C_{2}\frac{dv_{2}}{dt}=i-f_{2}(v_{2})\;L\frac{di}{dt}=v_{bias}-v_{1}-v_{2}-R\cdot i$$

where the state vector is **x** = (*v*_1_, *v*_2_, *i*)^T^, *C*_1_ and *C*_2_ is parasitic capacitance of first and second RTD, *L* and *R* is summarized (RTDs are connected in series) lead inductance and resistance, respectively. Details about modeling of high frequency RTD including typical values of parasitic elements can be found in [25]. We can express both nonlinear functions (for *k* = 1, 2) as:

(2) $$f_{k}(x)=a_{k}{\left(x-d_{k}\right)}^{3}+b_{k}(x-d_{k})+c_{k}$$

Thus, AVC of each RTD is a cubic polynomial that should form an N-type curve with a negative segment. Fixed points *x_e_* are all solutions of the problem *d***x**/*dt* = 0. For further simplicity, let’s assume that *R* = 0 Ω. We can determine global conditions and position for its existence within state space as each solution of system of the nonlinear algebraic equations, namely:

(3) $$\begin{array}{c} a_{1}{\left(x_{e}-d_{1}\right)}^{3}+b_{1}(x_{e}-d_{1})+c_{1}=a_{2}{\left(V_{bias}-x_{e}-d_{2}\right)}^{3}+b_{2}(V_{bias}-x_{e}-d_{2})+c_{2}\\ y_{e}=V_{bias}-x_{e}\\ z_{e}=a_{1}{\left(x_{e}-d_{1}\right)}^{3}+b_{1}(x_{e}-d_{1})+c_{1} \end{array}$$

Vector field geometry depends on the eigenvalues; i.e., roots of a characteristic polynomial. It can be calculated as det(*s*·**E**–**J**) = 0 where **E** is the unity matrix and **J** is the Jacobi matrix:

(4) $$J=\left(\begin{array}{ccc} -b_{1}-3a_{1}{\left(x_{e}-d_{1}\right)}^{2} & 0 & 1\\ 0 & -b_{2}-3a_{2}{\left(y_{e}-d_{2}\right)}^{2} & 1\\ -1 & -1 & 0 \end{array}\right).$$

Characteristic polynomial in symbolical form becomes:

(5) $$s^{3}+\{3a_{2}V_{bias}^{2}-6a_{2}V_{bias}(x_{e}+d_{2})+b_{1}+b_{2}+3a_{1}(x_{e}^{2}+d_{1}^{2})+3a_{2}(x_{e}^{2}+d_{2}^{2})-\;6x_{e}(a_{1}d_{1}-a_{2}d_{2})\}s^{2}\{[a_{1}a_{2}(9d_{1}^{2}-18d_{1}x_{e}+9x_{e}^{2})+3b_{1}a_{2}]V_{bias}^{2}+[a_{1}a_{2}(36d_{1}x_{e}^{2}-\;18d_{1}^{2}d_{2}-18d_{1}^{2}x_{e}+36d_{1}d_{2}x_{e}-18x_{e}^{3}-18d_{2}x_{e}^{2})-6b_{1}a_{2}x_{e}-6b_{1}a_{2}d_{2}]V_{bias}+b_{1}b_{2}+\;3a_{1}d_{1}^{2}b_{2}+3b_{1}a_{2}d_{2}^{2}+3a_{1}b_{2}x_{e}^{2}+3b_{1}a_{2}x_{e}^{2}-6a_{1}d_{1}b_{2}x_{e}+a_{1}a_{2}(9x_{e}^{4}-\;18d_{1}x_{e}^{3}-18d_{2}x_{e}^{3}+9d_{1}^{2}d_{2}^{2}+9d_{1}^{2}d_{e}^{2}+9d_{2}^{2}d_{e}^{2}-36d_{1}d_{2}x_{e}^{2}-18d_{1}d_{2}^{2}x_{e}+18d_{1}^{2}d_{2}x_{e})+\;2\}s+b_{1}+b_{2}+3V_{bias}^{2}a_{2}+3a_{1}d_{1}^{2}+3a_{2}d_{2}^{2}+3x_{e}^{2}(a_{1}+a_{2})-6V_{bias}a_{2}(d_{2}+a_{2}x_{e})-\;6x_{e}(a_{1}d_{1}-a_{2}d_{2})=0,$$

Obviously, symbolical expressions for the individual eigenvalues are very complicated and cannot further contribute to the better understanding of a vector field configuration and chaos evolution; check well-known Cardan rules. In situation, where *x_e_* and *y_e_* coordinate of equilibrium point is close to offset voltages represented by *d*_1_ and *d*_2_, characteristic polynomial simplifies into the relation:

(6) $$s^{3}+\left(b_{1}+b_{2}\right)s^{2}+\left(b_{1}b_{2}+2\right)s+b_{1}+b_{2}=0,$$

and the eigenvalues depend only on linear part of polynomial approximation of AVCs of RTDs.

Until very recently, analysis of MVMS was focused only on a high-frequency modeling of RTDs, influence of a pulse driving force on overall stability [26] and global dynamics [27] and specification of the boundary planes [28]. However, existence of chaos has not been uncovered and examined.

## 3. Numerical Results and Discussion

Numerical values of MVMS parameters can be obtained by the optimization technique described in [29]. In this case, the mathematical model of MVMS was considered piecewise-linear. Such kind of a vector field allows better understanding of chaos evolution, allows partial analytic solution and makes linear analysis generally more powerful. However, situation when AVCs of both RTDs are approximated by the polynomial functions is closer to reality. Thus, our problem stands as follows: couple of three-segment piecewise-linear functions needs to be transformed into the cubic (or higher-order if necessary) polynomial functions without losing robust chaotic solution having numerically close metric fractal dimension (Kaplan-Yorke is preferred over capacity because of rapid and precise calculation). For more details, readers should consult [30] where the inverse problem has been successfully solved. Finally, chaotic attractor like the so-called Rossler attractor [31] was localized.

Searching within smooth vector field (1) and by considering default normalized values *C*_1_ = 11 F, *C*_2_ = 37 F, *L* = 100 mH and *R* = 0 Ω leads to the following optimal values of the cubic polynomials (2):

(7) $$a_{1}=648.5,\;b_{1}=-23.1,\;c_{1}=-0.13,\;d_{1}=0.3,\;a_{2}=58.1,\;b_{2}=-21.9,\;c_{2}=-0.65,\;d_{2}=0.5.$$

**Figure 2:**
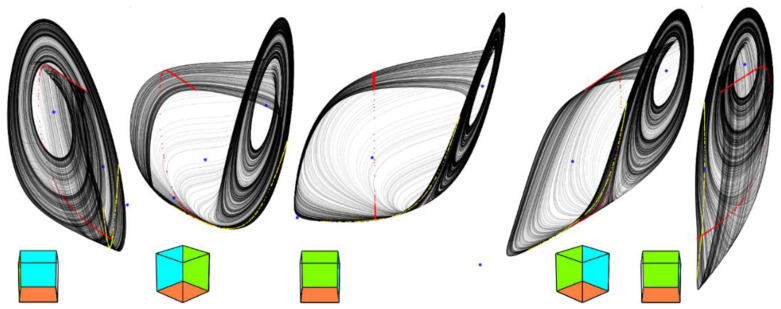
Three-dimensional perspective views on a typical chaotic attractor generated by polynomial MVMS for the initial conditions **x**_0_ = (0.1, 0.3, 0)^T^; Poincaré sections in planes shifted by the offsets of polynomial functions: *x* = *c*_1_ (red), *y* = *c*_2_ (yellow); fixed point of the flow (blue dots), state space rotation (no deformations of axis system). Numerical integration with final time 10^4^ and time step 0.01.

**Figure 3:**
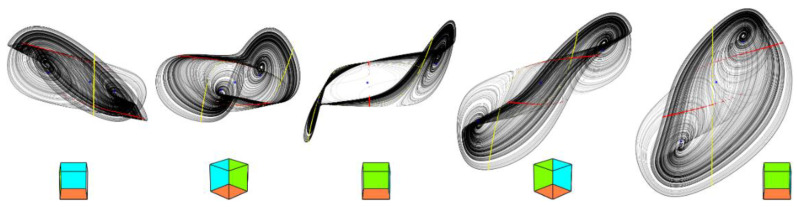
Three-dimensional perspective views on chaotic attractor with increased entropy generated by polynomial MVMS for the initial conditions **x**_0_ = (0.1, 0.3, 0)^T^; Poincaré sections in planes shifted by offsets of polynomial functions: *x* = *c*_1_ (red), *y* = *c*_2_ (yellow); fixed point of the flow (blue dots), state space rotation (no deformations of axis system). Calculation with final time 10^4^ and time step 0.01.

**Figure 4:**
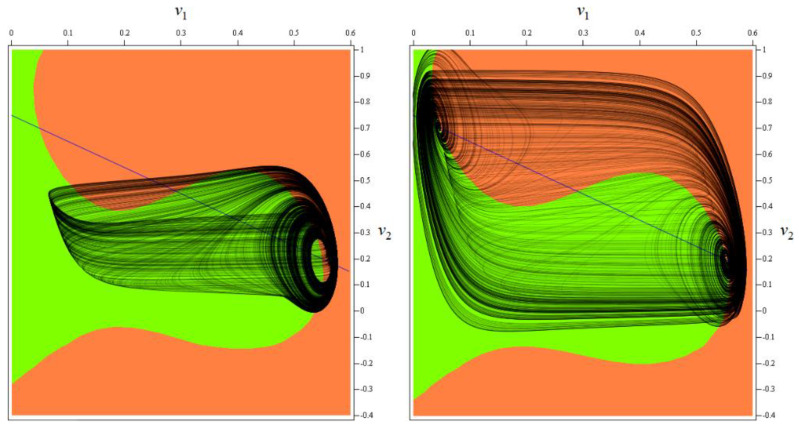
Important geometric structures located within state space: *v*_1_–*v*_2_ plane projection of function *f*_1_(*v*_1_) (orange), function *f*_2_(*v*_2_) (green) and *v*_1_ = *V_bias_* − *v*_2_ (blue line), intersections of these planes are fixed points of dynamical flow. Discovered chaotic attractor put into the context of vector field.

**Figure 5:**
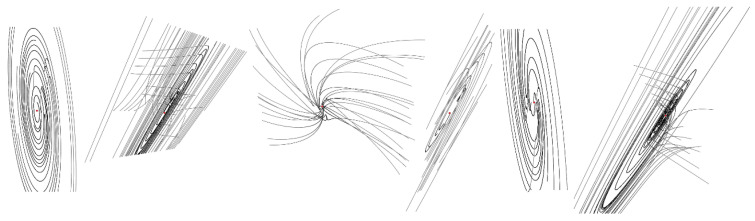
Graphical visualization of a dynamical behavior near the fixed points (red dots): equilibrium *x_e_* = 0.046 (left two images), for fixed point located at *x_e_* = 0.292 (middle two plots) and fixed point with *x_e_* = 0.55 (right two state portraits).

**Figure 6:**
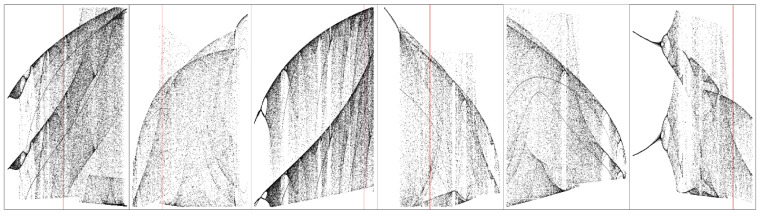
Gallery of one-dimensional bifurcation diagrams calculated with respect to the polynomial approximation of AVCs of RTDs; individual plots from left to right: horizontal axis represented by parameter range *a*_1_ ∈ (600, 700) calculated with step Δ = 0.1; parameter range *b*_1_ ∈ (−30, 0) established with step Δ = 0.01; parameter range *c*_1_ ∈ (−1.5, 0) together with step Δ = 0.001; parameter *a*_2_ ∈ (30, 100) with step Δ = 0.1; parameter *b*_2_ ∈ (−25, −20) together with step Δ = 0.01 and parameter *c*_2_ ∈ (−4, 0) with step Δ = 0.01.

Adopting these values and fourth-order Runge-Kutta numerical integration process we get the reference chaotic orbits provided in Figure 2. Accordingly to Equation (3) we have three fixed points: first located in position *x_e_* = (0.046, 0.704, −4.768)^T^ and characterized by eigenvalues *λ*_1_ = −101.241, *λ*_2_ = 0.062, *λ*_3_ = 13.915, second equilibria in position *x_e_* = (0.292, 0.458, 0.043)^T^ having set of the eigenvalues *λ*_1_ = 0.09, *λ*_2_ = 22.97, *λ*_3_ = 21.72 and *x_e_* = (0.55, 0.2, 4.299)^T^ with local behavior given by eigenvalues *λ*_1_ = −99, *λ*_2_ = 0.13, *λ*_3_ = 7.146. This is an interesting geometric configuration of the vector field: a funnel-type strange attractor generated by saddle-node equilibria with the stability index 1, 0 and 0; saddle-focuses are completely missing. Developed optimization algorithm can be utilized to maximize unpredictability of a dynamical flow and increase system entropy. Starting with values (7) the following set of numerical values was obtained:

(8) $$a_{1}=680,\;b_{1}=-23,\;c_{1}=-0.13,\;d_{1}=0.3,\;a_{2}=55,\;b_{2}=-25,\;c_{2}=-0.65,\;d_{2}=0.5,$$

leading to a double-hook [12] like chaotic attractor visualized by means of Figure 3. The position of the first fixed point changes slightly to *x_e_* = (0.045, 0.705, −5.48)^T^ as well as the corresponding eigenvalues *λ*_1_ = −109.037, *λ*_2_ = 0.049, *λ*_3_ = 13.915. The second equilibrium moves into position *x_e_* = (0.29, 0.46, 0.089)^T^ and possesses the set of eigenvalues *λ*_1_ = 0.084, *λ*_2_ = 22.764, *λ*_3_ = 24.817. Finally, the last fixed point moves towards position *x_e_* = (0.554, 0.196, 5.31)^T^ where the local behavior will be given by the eigenvalues *λ*_1_ = −109.953, *λ*_2_ = 0.085, *λ*_3_ = 10.596. The mentioned equilibria together with important state space sections are provided in Figure 4. Note that local geometries of a vector field are not affected since, in a closed neighborhood of the fixed points, dynamical movement is given by three eigenvectors as in the case of a funnel chaotic attractor. Also note that one direction of the flow (along the eigenvector associated with *λ*_1_) is strongly attracting; this nature is evident from Figure 5. Interesting fragments of the bifurcation diagrams are depicted in Figure 6. 

Here, transient motion has been removed and plane *x* = *d*_1_ has been used for individual slices. These are plotted against parameters *a_k_*, *b_k_* and *c_k_* of polynomial approximations of AVC of *k*-th RTD. Parameters *a*_1_ and *a*_2_ can burn or bury chaotic attractor since these affect eigenvalues in “outer” parts of the active vector field (given by size of the state attractor) while geometry around “middle” fixed point remains almost unchanged.

**Figure 7:**
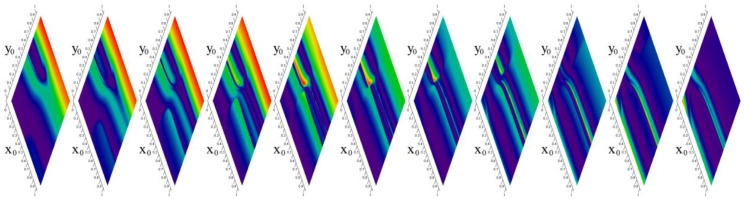
Rainbow-scaled contour plots of short-time evolution of MVMS energy for the state space slices forming unity cube (left to right): *z*_0_ = −5, *z*_0_ = −4, *z*_0_ = −3, *z*_0_ = −2, *z*_0_ = −1, *z*_0_ = 0, *z*_0_ = 1, *z*_0_ = 2, *z*_0_ = 3, *z*_0_ = 4, *z*_0_ = 5.

Figure 7 shows numerical calculation of a gained energy with small time step with respect to the state space location; red regions mark large increment while dark blue stands for a small evolution.

**Figure 8:**
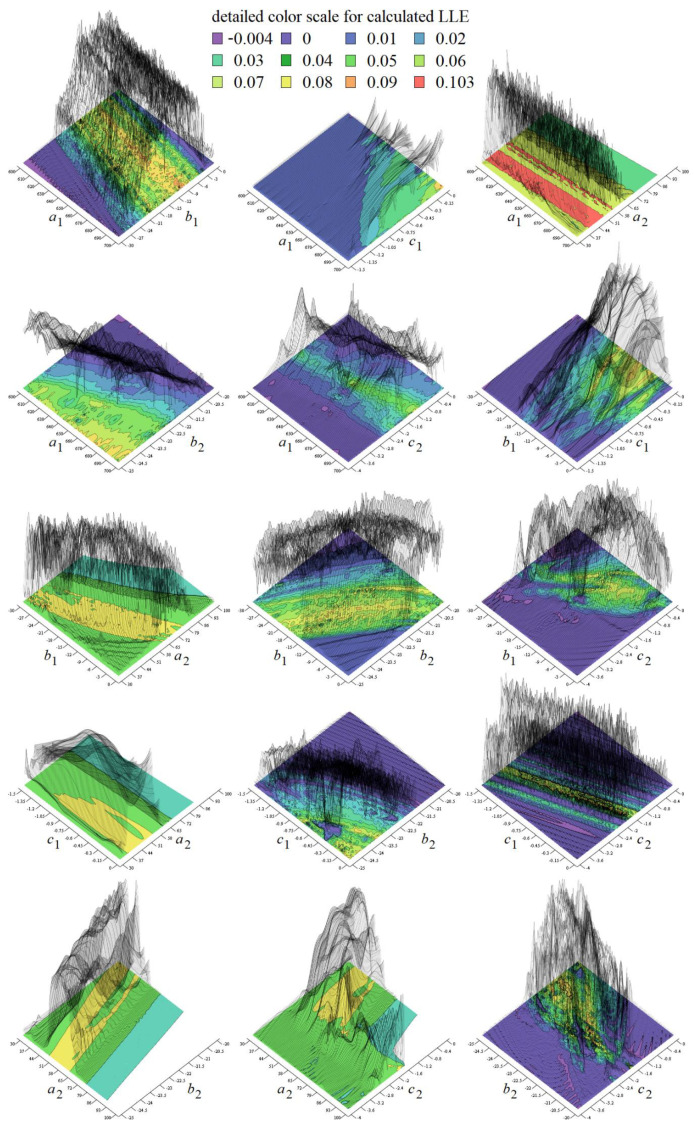
Gallery of the rainbow-scaled surface-contour plots of LLE as functions of two parameters; vertical range dedicated for LLE is −0.004 to 0.103, counting of plots from left to right and up to down: *a*_1_–*b*_1_, *a*_1_–*c*_1_, *a*_1_–*a*_2_, *a*_1_–*b*_2_, *a*_1_–*c*_2_, *b*_1_–*c*_1_, *b*_1_–*a*_2_, *b*_1_–*b*_2_, *b*_1_–*c*_2_, *c*_1_–*a*_2_, *c*_1_–*b*_2_, *c*_1_–*c*_2_, *a*_2_–*b*_2_, *a*_2_–*c*_2_, *b*_2_–*c*_2_.

As stated before, chaotic solution is sensitive to the changes of parameters *a_k_*, *b_k_*, *c_k_* and *d_k_*, where *k* = 1, 2. If we consider these values variable we create eight-dimensional hyperspace in which chaos alternates with periodic orbits or fixed-point solution. Two-dimensional subspaces hewed-out from such hyperspace are provided in Figure 8. Each graph is composed of 101 × 101 = 10,201 points, calculation routine deals with time interval t ∈ (100, 10^4^), random initial conditions inside basin of attraction and Gram-Smith orthogonalization [32]. Here, dark blue represents a trivial solution, light blue a limit cycle, green color stands for weak chaos and yellow marks strong chaotic behavior. Note that LLE for set of values (7) can be found in each visualized plot. 

Maximal merit of LLE is 0.089 for a value set (7) and 0.103 for (8) and associated Kaplan–Yorke dimension [33] is 2.016 and 2.021 respectively. Specification of sufficiently large “chaotic” area is important also from practical viewpoint; as will be clarified later. 

## 4. Circuitry Realization of MVMS-Based Chaotic Oscillators

**Figure 9:**
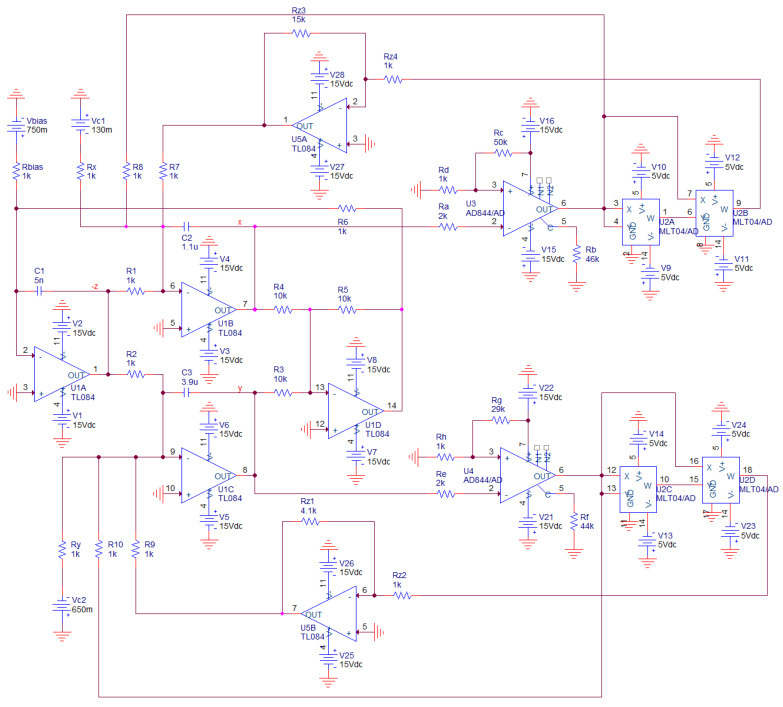
Chaotic oscillator designed to audio band based on integrator block schematic associated with mathematical model of MVMS, numerical values of passive network components are included.

Design of analog equivalent circuit is common way how to prove existence of structurally stable strange attractors within the dynamics of a prescribed set of ordinary differential equations. Realization of such the so-called chaotic oscillators is a simple and straightforward task that we can solve by using several approaches [34,35,36,37,38], both using discrete components and in integrated form. A favorite method that allows us to utilize commercially available active elements follows the concept of analog computers. Thus, only three building blocks are required for circuit construction: inverting summing integrator, summing amplifier and, in the case of a polynomial nonlinearity, four-segment analog multiplier. Fully analog circuit implementation is shown in Figure 9. 

Note that this circuit synthesis requires many active devices: two TL084, two AD844 and a single four channel four quadrant analog multiplier MLT04. Supply voltage is symmetrical ±15 V but, for MLT04, this voltage is lowered to ±5 V. Majority of the analog realizations of the chaotic systems with a polynomial nonlinearity utilize AD633, i.e., single channel multiplier. It is possible also in our case but with the cost of eight active devices. Dynamical range for correct operation of MLT04 is only ±2 V. However, prescribed strange attractor is smaller in *v*_1_–*v*_2_ dimension. Advantage of this circuit is that individual MVMS parameters can be adjusted independently using potentiometers. Theoretically, using different decomposition of the polynomial functions, total number of the active elements can be lowered to four. Proposed chaotic oscillator is uniquely described by the following set of the differential equations:

(9) $$\frac{dv_{1}}{dt}=-\frac{1}{C_{2}}\left[-\frac{v_{3}}{R_{1}}-\frac{R_{b}}{R_{a}}\left(v_{1}-\frac{R_{d}}{R_{d}+R_{c}}V_{cc}\right)\left\{\frac{1}{R_{8}}-\frac{R_{z3}}{R_{z4}}{\left(\frac{R_{b}}{R_{a}}\right)}^{3}K^{2}\left(v_{1}-\frac{R_{d}}{R_{d}+R_{c}}V_{cc}\right)\frac{1}{R_{7}}\right\}-\frac{V_{c1}}{R_{x}}\right]\;\frac{dv_{2}}{dt}=-\frac{1}{C_{3}}\left[-\frac{v_{3}}{R_{2}}-\frac{R_{f}}{R_{e}}\left(v_{2}-\frac{R_{h}}{R_{h}+R_{g}}V_{cc}\right)\left\{\frac{1}{R_{10}}-\frac{R_{z1}}{R_{z2}}{\left(\frac{R_{f}}{R_{e}}\right)}^{3}K^{2}\left(v_{2}-\frac{R_{h}}{R_{h}+R_{g}}V_{cc}\right)\frac{1}{R_{9}}\right\}-\frac{V_{c2}}{R_{y}}\right]\;\frac{dv_{3}}{dt}=\frac{1}{C_{1}}\left[-\frac{R_{5}}{R_{6}}\left(\frac{v_{1}}{R_{4}}+\frac{v_{2}}{R_{5}}\right)+\frac{V_{bias}}{R_{bias}}\right]\;$$

where *V_C_*_1_, *V_C_*_2_ and *V_bias_* are independent dc voltage sources and *K* = 0.4 is internally-trimmed scaling constant of the analog multiplier cells MLT04. Fundamental time constant of this chaotic oscillator is chosen to be *τ* = *R*·*Cv* = 10^3^·10^−7^ = 100 μs.

**Figure 10:**
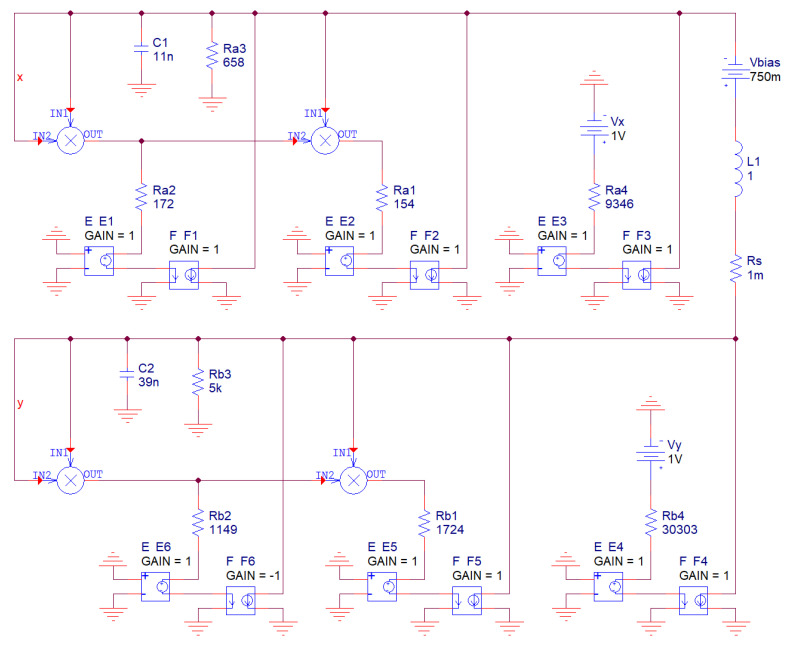
Chaotic system obtained directly from fundamental MVMS with ideal multipliers and ideal second-generation current-conveyors, numerical values of the circuit components are included.

Of course, it is also possible to build both analog networks provided in Figure 1 directly. Instead of nonlinear two-ports we must construct a couple of resistors with polynomial AVC; systematic design towards these network elements can be found in [39,40]. Circuitry realization of original MVMS with state vector **x** = (*v*_1_, *v*_2_, *i*)^T^ is demonstrated by means of Figure 10; i.e., the state variables are voltages across grounded capacitors and current flowing through the inductor. Note that both polynomial function (2) need to be rewritten into the form *i* = *f*(*v*) = *a*·*v*^3^ + *b*·*v*^2^ + *c*·*v* + *d*. Thus, a new set of the dimensionless ordinary differential equations to be implemented as lumped analog electronic circuit as follows:

(10) $$\frac{dx}{dt}=-z-648\cdot x^{3}-583\cdot x^{2}-152\cdot x-10.7\;\frac{dy}{dt}=z-58\cdot y^{3}+87\cdot y^{2}-20\cdot y-3.3\;\frac{dz}{dt}=x-y+0.75\;$$

Now assume that impedance and frequency norm are 10^5^ and 10^4^, respectively. Such values lead to the nominal inductance 1H. This simplified concept of chaotic oscillators is given in Figure 10 and described by following set of the ordinary differential equations:

(11) $$C_{1}\frac{dv_{1}}{dt}+i_{L}+\frac{v_{1}^{3}}{R_{a1}}+\frac{v_{1}^{2}}{R_{a2}}+\frac{v_{1}}{R_{a3}}+\frac{V_{X}}{R_{a4}}=0\;C_{2}\frac{dv_{2}}{dt}+\frac{v_{2}^{3}}{R_{b1}}+\frac{v_{2}}{R_{b3}}+\frac{V_{Y}}{R_{b4}}=i_{L}+\frac{v_{2}^{2}}{R_{b2}}\;L\frac{di_{L}}{dt}+R_{S}i_{L}+v_{2}=v_{1}+V_{bias}$$

where a small value *R_S_* can still model lead to resistances of both RTDs that are parts of MVMS in Figure 1.

**Figure 11:**
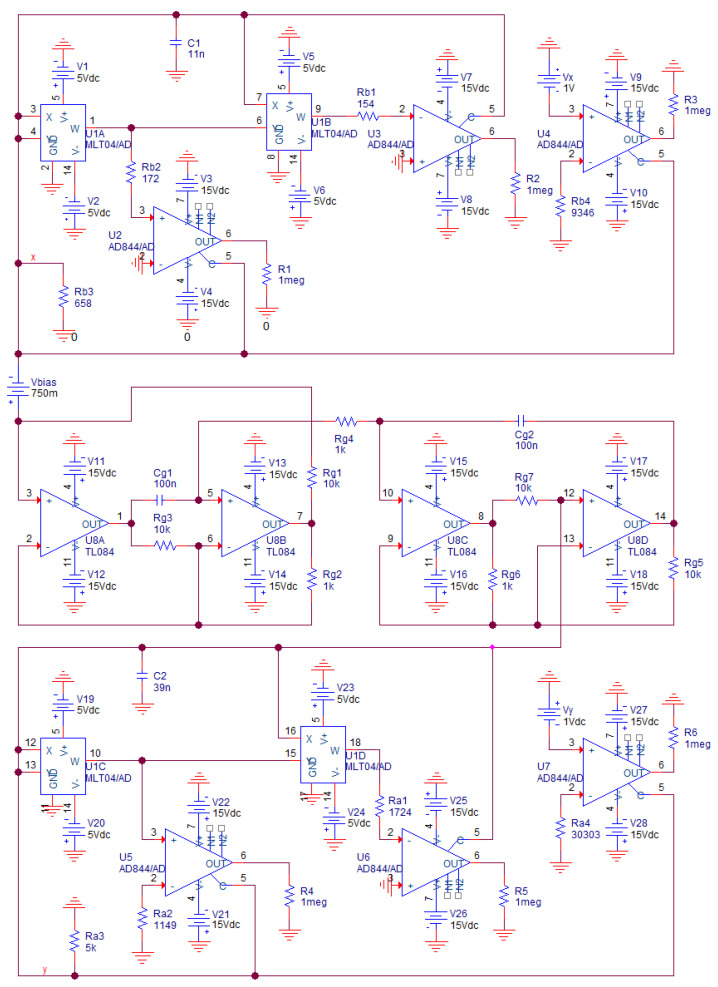
Chaotic system obtained directly from fundamental MVMS network with floating synthetic inductor, numerical values of the passive circuit components are included, ready for verification.

Note that this kind of realization utilizes second generation current conveyors implemented by using ideal voltage-controlled voltage-source E and current-controlled current-source F. A positive variant of this active three-port element is commercially available as the AD844 while a negative variant is EL2082 (only one negative device is required). In practice, the inductor should be substituted by the synthetic equivalent; i.e., active floating gyrator (Antoniou’s sub-circuit) with a capacitive load, check Figure 11. In this case, the number of the active elements raises to eight: a single TL084, six AD844s and a single MLT04. Dynamical behavior is uniquely determined by the following mathematical model:

(12) $$\frac{dv_{1}}{dt}=\frac{1}{C_{1}}\left[-i-\frac{K^{2}v_{1}^{3}}{R_{a1}+R_{in}}-\frac{Kv_{1}^{2}}{R_{a2}+R_{in}}-\frac{v_{1}}{R_{a3}}-\frac{V_{x}}{R_{a4}+R_{in}}\right]\;\frac{dv_{2}}{dt}=\frac{1}{C_{2}}\left[-i-\frac{K^{2}v_{2}^{3}}{R_{b1}+R_{in}}-\frac{Kv_{2}^{2}}{R_{b2}+R_{in}}-\frac{v_{2}}{R_{b3}}-\frac{V_{y}}{R_{b4}+R_{in}}\right]\;\frac{di}{dt}=\frac{R_{g2}}{R_{g1}R_{g3}R_{g4}C_{g1}}\left[v_{1}-v_{2}+V_{bias}\right]$$

where *R_in_* represents input resistance of current input terminal of AD844. Its typical value is 50 Ω and, due to the high values of the polynomial coefficients and in the case of small impedance norm chosen, it cannot be generally neglected. On the other hand, we must avoid output current saturation of each AD844 and consider frequency limitations of each active device. Thus, choice of both normalization factors is always a compromise. Thanks to the symmetry inside floating Antoniou’s structure *R_g_*_5_ = *R_g_*_3_, *R_g_*_7_ = *R_g_*_1_, *R_g_*_6_ = *R_g_*_2_ and *C_g_*_2_ = *C_g_*_1_. Behavior of this chaotic oscillator is extremely sensitive to the working resistors connected in the nonlinear two-terminal devices. Thus, calculated values were specified by Orcad Pspice optimizer where fitness functions (several should be defined to create tolerance channel) are absolute difference between polynomials in (11) and actual input resistance of designed circuit. Corresponding dc sweep analysis were estimated for input voltages from 0 V to 2 V with step 10 mV.

**Figure 12:**
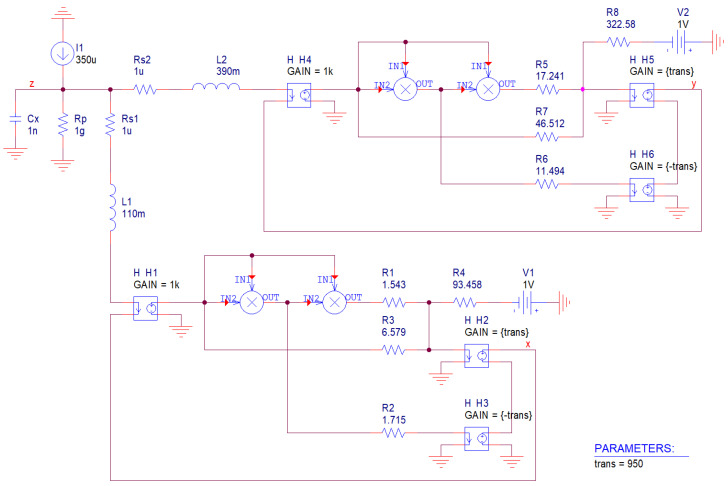
Hypothetic analog circuit realization dual to original MVMS network with ideal controlled sources: trans-resistance of output current-to-voltage conversion is considered as global parameter.

Last circuitry implementation is provided in Figure 12; namely dual network to the original MVMS. Impedance norm is chosen to be 10^3^ and frequency normalization factor is 10^5^. To get the reasonable values of the resistors further impedance rescaling is possible. Set of describing differential equations can be expressed as follows:

(13) $$\frac{di_{L1}}{dt}=\frac{1}{L_{1}}\left[-R_{s1}i_{L1}+r_{T2}\left\{\frac{{\left(r_{T1}i_{L1}\right)}^{3}}{R_{1}}+\frac{r_{T1}i_{L1}}{R_{3}}-\frac{V_{1}}{R_{4}}\right\}-r_{T3}\frac{{\left(r_{T1}i_{L1}\right)}^{2}}{R_{2}}\right]\;\frac{di_{L2}}{dt}=\frac{1}{L_{2}}\left[-R_{s2}i_{L2}+r_{T5}\left\{\frac{{\left(r_{T4}i_{L2}\right)}^{3}}{R_{5}}+\frac{r_{T4}i_{L2}}{R_{7}}-\frac{V_{2}}{R_{8}}\right\}-r_{T6}\frac{{\left(r_{T4}i_{L2}\right)}^{2}}{R_{6}}\right]\;\frac{dv_{Z}}{dt}=\frac{1}{C_{X}}\left(-\frac{v_{Z}}{R_{p}}-i_{L1}-i_{L2}+I_{1}\right)$$

where *r_Tk_* is trans-resistance of *k*-h ideal current-controlled voltage-source and *I*_1_ is independent dc current source.

Polynomial nonlinearity is implemented by ideal multiplication using block MULT. Current flowing through both inductors can be sensed via small resistor *R_S_*_1_ and *R_S_*_2_ respectively. However, these resistors represent error terms that are inserted into describing differential equations; similarly as a parasitic shunt resistor *R_p_*. Such parasitic properties change global dynamics, can boost system dimensionality (not in this situation) and desired chaotic behavior can eventually disappear.

Unfortunately, active elements where output voltage is controlled by input current are not off-the-shelf components. However, trans-resistance amplifier can be constructed using single standard operational amplifier and feedback resistor. To do this, input is fed directly to − terminal, node + is connected to ground, resistor between − and OUT. Node OUT also represents output of a designed transimpedance amplifier. Equivalently, the AD844 can also do the trick. Input can be connected to − terminal, ground to + terminal, resistor between C and ground and output to OUT. Considering the latter case, number of the active devices becomes seven: one MLT04 and six AD844.

Colored plots provided in Figure 8 demonstrate that region of chaos around discovered values (7) is wide enough to provide a structurally stable strange attractor; both funnel and double-scroll type. The same analysis was performed for set (8); this strange attractor should be also observable.

## 5. Circuit Simulation, Experimental Verification and Comparison

**Figure 13:**
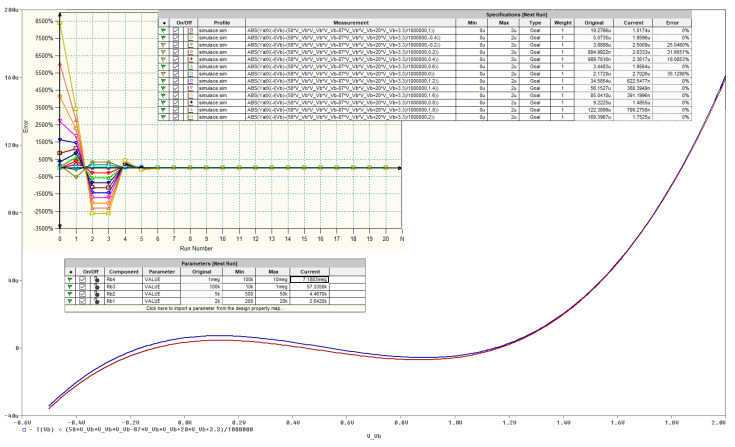
Orcad Pspice optimization toolbox: definitions of the objective functions and requested maximal errors (upper right field), error graph (upper left), new values of resistors (middle table).

As mentioned before, AVC of both nonlinear resistors in Figure 11 should be as precise as possible to reach desired state space attractor. To fulfil this requirement, build-in Orcad Pspice optimizer can be adopted; see Figure 13 where optimal AVC of one nonlinear two-terminal device is reached.

Of course, the operational regime of this nonlinear resistor needs to be limited; in our case to input voltages into range starting with −0.5 V and ending with 2 V. Note that up to thirteen fitness functions were supposed to cover predefined operational range. Also note that optimization was stopped while some objective functions still have nonzero error (expressed in terms of the differential percentages). Important is the matter of similarity between simulated and numerically integrated strange attractor.

Implementation of the chaotic oscillator based on the integrator block schematic is more robust; i.e., the desired strange attractor is less vulnerable to the passive component matching: fabrication series (E12) and component tolerances (1%) were considered for design. We are also experiencing superior parasitic properties of the voltage-mode active elements: both integrated circuits TL084 and MLT04 have very high input and very low output resistances. Values of resistors that form external network connected to AD844 were chosen such that parasitic input and output impedances can be neglected. This is the main reason why this kind of MVMS realization was picked and forwarded into practical experiments and undergoes laboratory measurement.

Generally, parasitic properties of the active elements play important role in a design process of analog chaotic oscillators and, of course, should be minimized. Besides input and output impedances also roll-off effects of transfer constants need to be analyzed. Several publications have been devoted to reveal and study problems associated with the parasitic properties of the specific active devices and how these affect global dynamics; for example [41].

**Figure 14:**
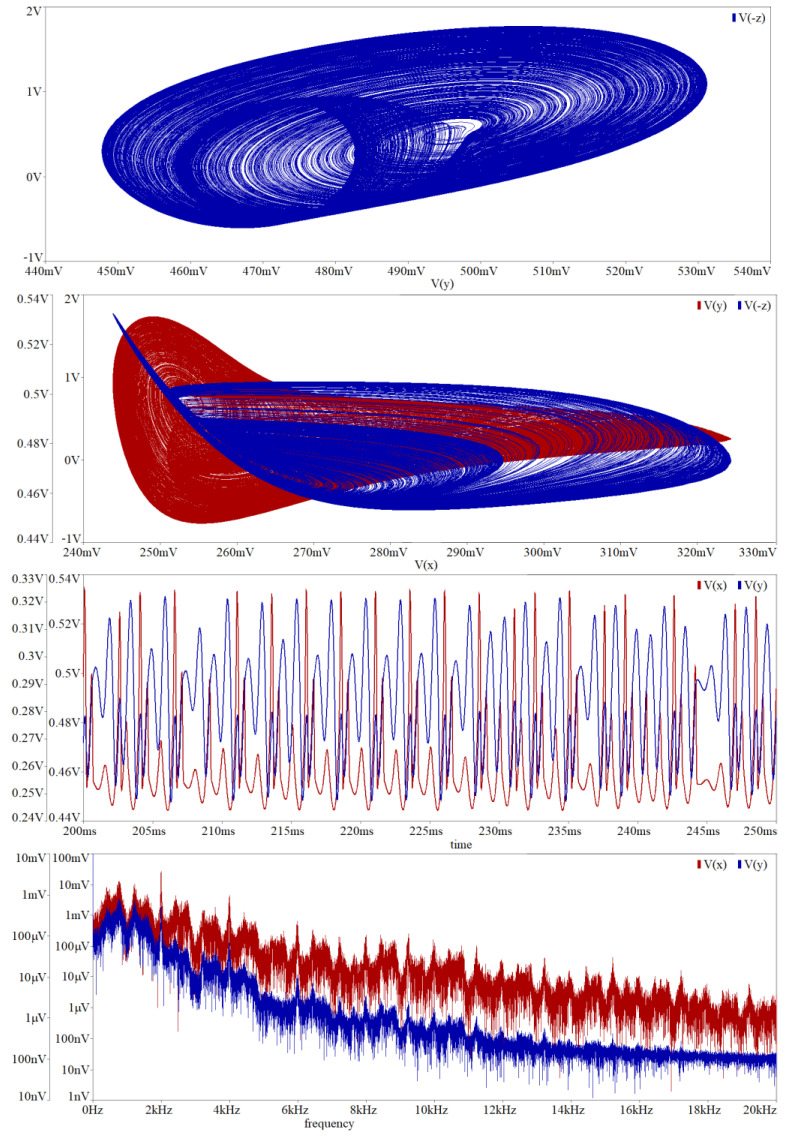
Orcad Pspice circuit simulation associated with chaotic circuit given in Figure 9: selected plane projection *v*_3_–*v*_2_ (blue, upper graph), *v*_2_–*v*_1_ (red) and *v*_3_–*v*_1_ (blue) of the chaotic attractor, generated chaotic signal *v*_1_ (red) and *v*_2_ (blue) in the time domain, chaotic waveform *v*_1_ (red) and *v*_2_ (blue) in the frequency domain. Note that significant frequency components are concentrated in audio range.

**Figure 15:**
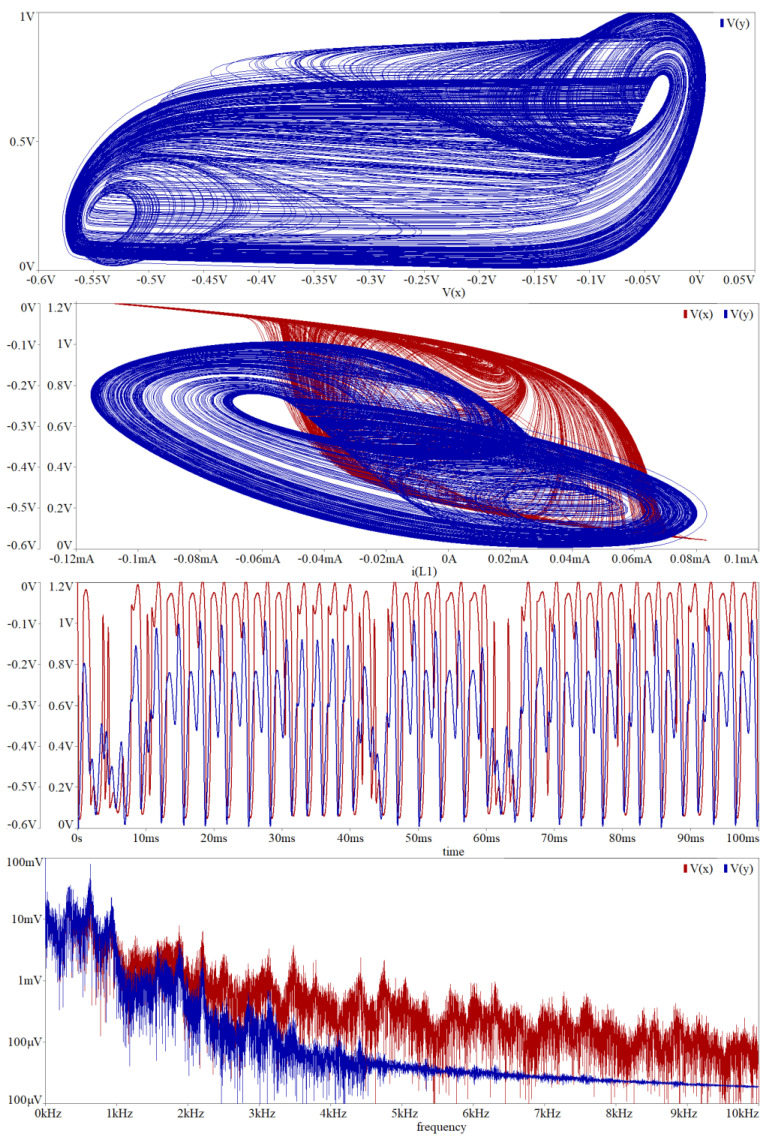
Orcad Pspice circuit simulation associated with network given in Figure 10: selected plane projection *v*_1_–*v*_2_ (blue, upper plot), *v*_1_–*i*_L_ (red) and *v*_2_–*i*_L_ (blue) of chaotic attractor, generated signal *v*_1_ (red) and *v*_2_ (blue) in time domain, chaotic waveform *v*_1_ (red) and *v*_2_ (blue) in frequency domain.

**Figure 16:**
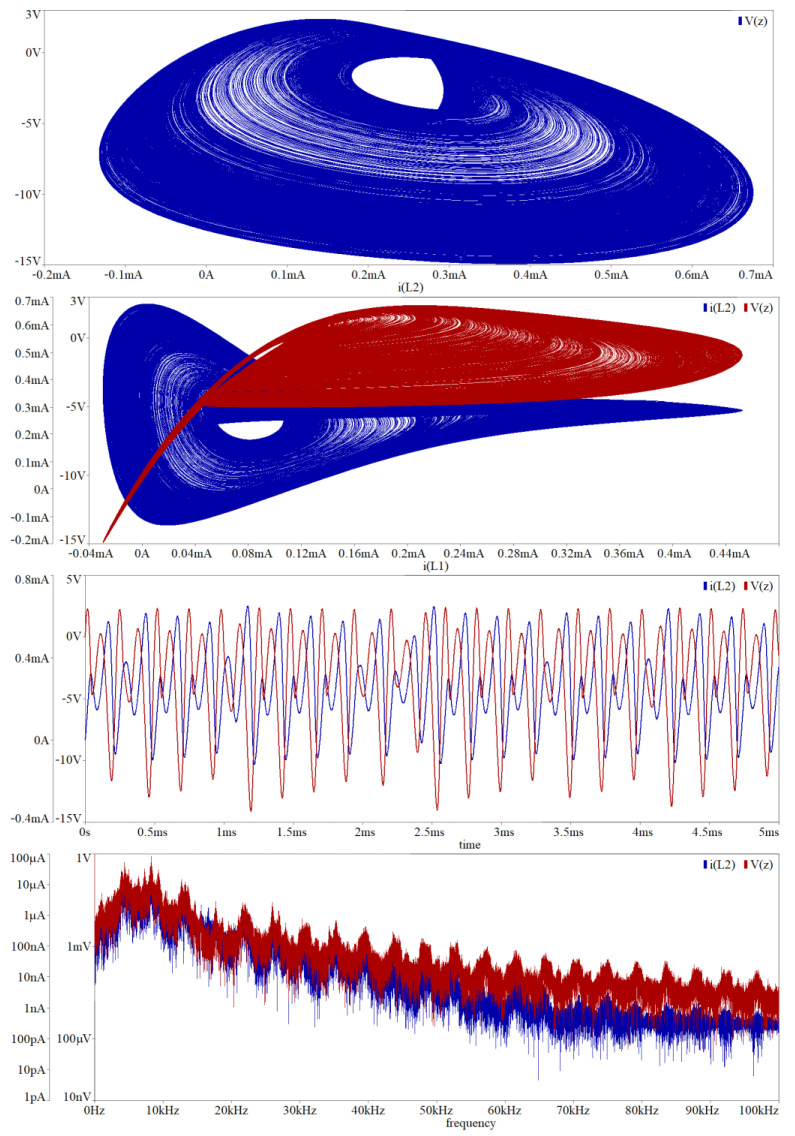
Orcad Pspice circuit simulation associated with network given in Figure 10: plane projection *v_Z_*–*i_L_*_2_ (blue, upper plot), *i_L_*_2_–*i_L_*_1_ (red) and *v_Z_*–*i_L_*_1_ (blue) of the observed strange attractor, generated chaotic signal *i_L_*_2_ (red) and *v_Z_* (blue) in the time domain, chaotic waveform *i_L_*_2_ (red) and *v_Z_* (blue) in the frequency domain. Note that generated chaos is not affected by saturation levels of the active devices.

In simulation profile of transient response, final time was set to 1 s and maximal time step 10 μs. This setup is kept for each simulation mentioned in this section. Circuit simulation associated with Figure 9 is provided in Figure 14. To transform the funnel into a double-scroll chaotic attractor we should change value of the resistors *R_z_*_3_, *R_z_*_1_ and *R*_10_. Simulation results associated with direct realization of original MVMS provided in Figure 10 is demonstrated by means of Figure 15. Computer-aided analysis of chaotic oscillator with idealized controlled sources is showed in Figure 16.

**Figure 17:**
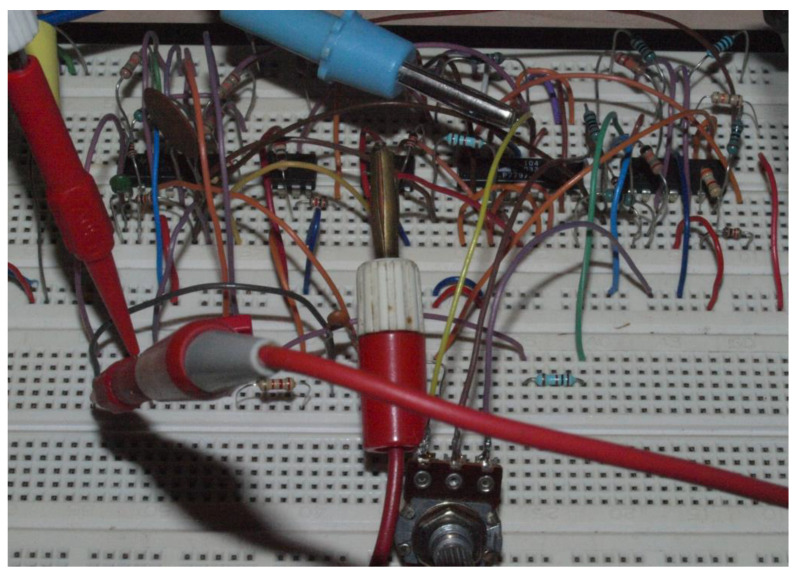
Practical realization of the integrator-based chaotic MVMS using bread-board.

**Figure 18:**
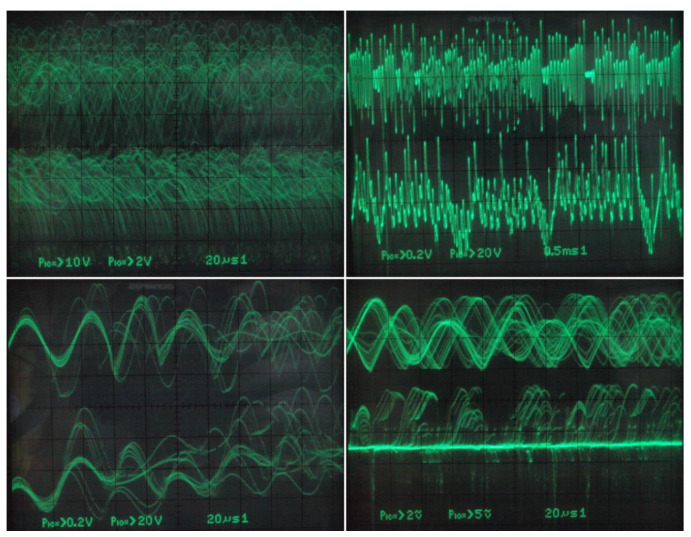
Selected chaotic waveforms in time domain generated by integrator-based MVMS.

Existence of observable strange attractors and numerically expected routing-to-chaos scenarios within dynamics of the fundamental MVMS has been proved experimentally; by its construction on the bread-board (see Figure 17) and consequent laboratory measurement using the analog oscilloscope. Due to its simpler realization and increased robustness, only circuitry illustrated by means of Figure 9 was decided for a real measurement. Selected chaotic waveforms in a time domain are provided by means of Figure 18. Independent voltage source *V_bias_* = 750 mV in series with *R_bias_* = 1 kΩ can be replaced by the positive supply voltage +5 V and the fixed series resistance *R_bias_* = 6.7 kΩ. Analogically, combinations *V_c_*_1_ = −130 mV, *R_x_* = 1 kΩ and *V_c_*_2_ = −650 mV, *R_y_* = 1 kΩ can be replaced by the negative supply voltage −5 V, *R_x_* = 38.5 kΩ and the same voltage −5 V, *R_y_* = 7.7 kΩ respectively. 

**Figure 19:**
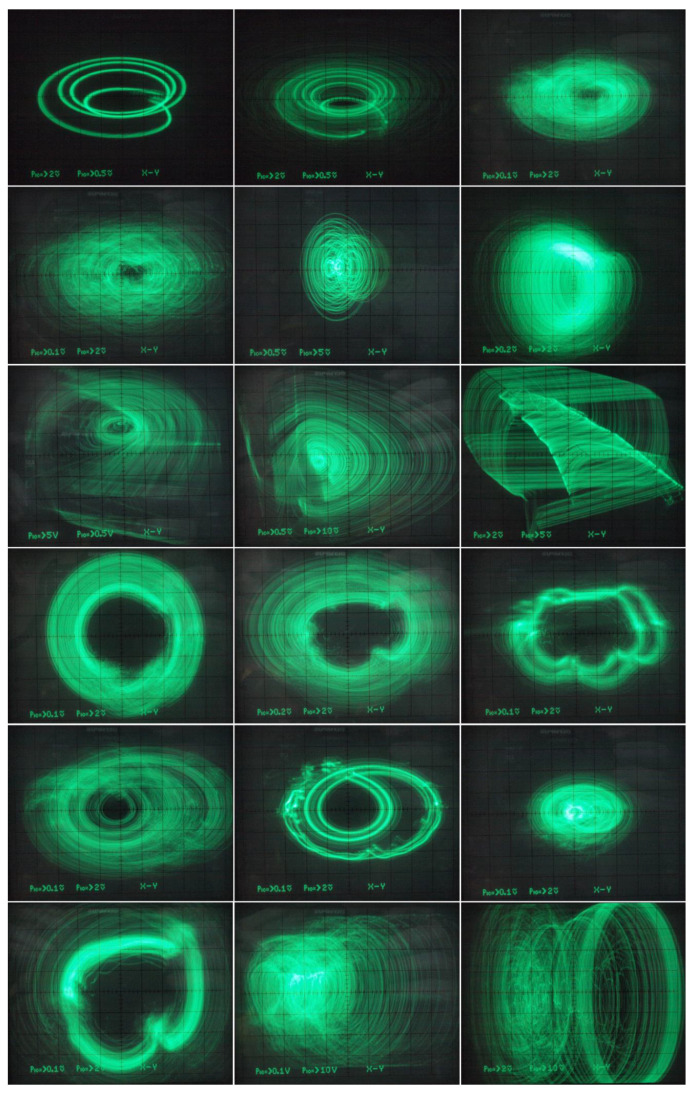
Selected *v*_3_–*v*_2_ plane projections measured of integrator-based MVMS realization, see text.

**Figure 20:**
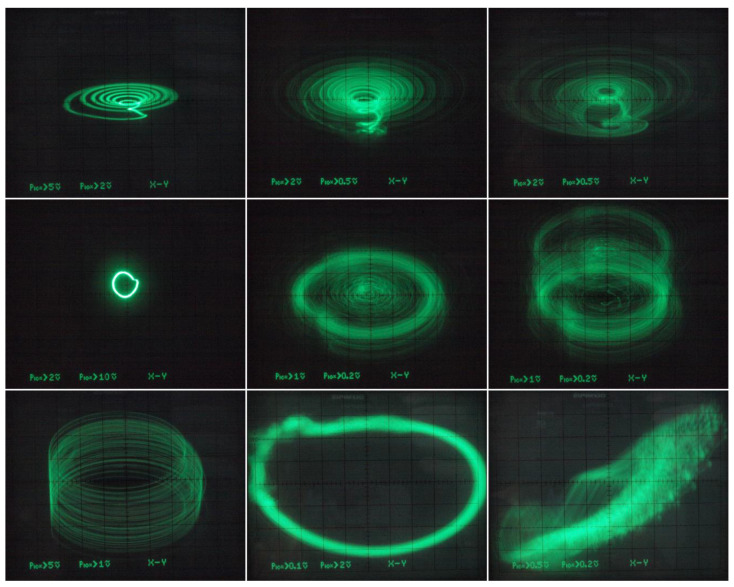
Few selected *v*_3_–*v*_1_ plane projections measured within dynamics of the integrator-based implementation MVMS, voltage sources *V_c_*_1_ and *V_c_*_2_ are swept, i.e., system parameters *c*_1_ and *c*_2_ are considered as variable parameters, change of the voltages starts within bifurcation diagrams provided by means of Figure 6 but finally goes far beyond it.

**Figure 21:**
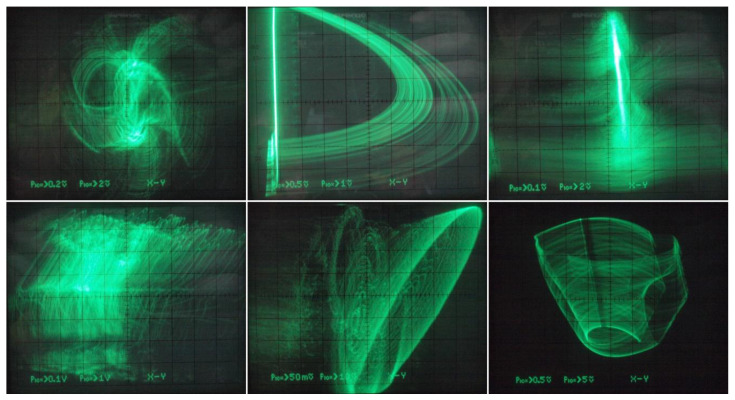
Gallery of interesting (still robust and real-time observable) strange attractors generated by the integrator-based realization of MVMS, different plane projections, see text for details.

Thus, there is no need to introduce three additional independent dc voltage sources into oscillator. However, to trace route-to-chaos scenarios, MVMS parameters *c*_1_ and *c*_2_ represented by voltages *V_c_*_1_ and *V_c_*_2_ have been considered as the variables. In practice, two voltage dividers based on potentiometers followed by two voltage buffers (remaining part of fifth integrated circuit TL084) did the trick. Since extreme values of *V_c_*_1_, *V_c_*_2_ voltages can be managed observed Monge projections given in Figure 19 (plane *v*_3_ vs. *v*_2_) originate both inside and outside prescribed bifurcation scheme pictured in Figure 6 (third and sixth plot). Of course, very small steps of the hand-swept parameters cannot be captured by the oscilloscope. Different plane projections, namely *v*_3_ vs. *v*_1_, are shown in Figure 20. The provided screenshots are centered on captured state attractors not the origin of the state coordinates. Of course, the mentioned plane projections are not in full one-to-one correspondence with the theoretical results given in Figure 14, simply because transfer functions of the nonlinear two-ports cannot be defined precisely using imperfect discrete resistors; it does not get better shape even if the working resistors are replaced by the standard hand potentiometers. Finally, the sequence of periodic and chaotic windows with continuous change of the individual coefficients of the polynomials has been confirmed; roughly proving the correctness of bifurcation diagrams in Figure 6. During laboratory experiments, unexpected and a very interestingly-shaped strange attractors were identified (see Figure 21). Unfortunately, numerical values of the mathematical model parameters were not found, and reference state trajectories cannot be created. Initial conditions need not to be imposed on the outputs of the inverting integrators (capacitors need not to be pre-charged) since neighborhood of zero belongs into the basin of attraction for both strange attractors.

## 6. Conclusions

Existence of robust chaotic attractors in the smooth vector field of MVMS have been demonstrated in this paper. This work represents a significant extension of the discoveries discussed in [29]. Both referred chaotic attractors are self-excited attractors; hidden attractors were not sought after and, in the case of analyzed MVMS dynamics, remain a mystery.

The proposed mathematical model: (1) together with the nonlinear functions (2) and parameters (7) or (8) can be also considered as a new chaotic dynamical system. This is still an up-to-date problem for many engineers and the topic of many recent scientific papers. However, algebraically much simpler dynamical systems that exhibit chaotic attractors exist; reading [42,43,44,45,46,47] is recommended.

Three different circuit realizations are presented. Each was verified by circuit simulation and the most robust implementation undergoes experimental measurement. Two designed oscillators utilize off-the-shelf active elements and can serve for various demonstrations.

The fundamental motivation of this work is to show that structurally stable strange attractors can be observed in smooth dynamical system naturally considered as nonlinear but with non-chaotic limit sets. However, designed autonomous chaotic oscillators can serve as the core circuits in many practical applications such as cryptography [48], spread-spectrum modulation techniques [49], useful signal masking [50,51], random number generators [52,53], etc.

This work also leaves several places for future research. For example, following interesting questions needs to be answered: Are there some hidden attractors? Are multi-scroll chaotic attractors observable if many RTD will be connected appropriately? Can MVMS generate chaos if values of the internal parameters are close to microelectronic memory cell fabricated in common technology?
